# Cell Biology of Pathogenic Protozoa and Their Interaction with Host Cells

**DOI:** 10.1155/2014/143418

**Published:** 2014-06-05

**Authors:** Marlene Benchimol, Juan C. Engel, Kevin S. W. Tan, Wanderley de Souza

**Affiliations:** ^1^Universidade Santa Úrsula, Rio de Janeiro, Brazil; ^2^Instituto de Biofísica Carlos Chagas Filho, Universidade Federal do Rio de Janeiro, Brazil; ^3^Instituto Nacional de Metrologia e Qualidade Industrial—Inmetro, Rio de Janeiro, Brazil; ^4^Instituto Nacional de Ciência e Tecnologia em Biologia Estrutural e Bioimagens-INBEB, Brazil; ^5^University of California, San Francisco-Pathology, California Institute for Quantitative Biosciences (QB3), UCSF-MC 2550-Rm. 501E, 1700 4th Street San Francisco, CA 94158-2330, USA; ^6^Laboratory of Molecular and Cellular Parasitology, Department of Microbiology, National University of Singapore, 5 Science Drive 2, Singapore 117545

The Kingdom Protista comprises a large number of eukaryotic microorganisms which are agents of important parasitic diseases. Some of these diseases, such as Chagas disease caused by* Trypanosoma cruzi*, are mainly restricted to the Latin American regions. Others, such as toxoplasmosis caused by* Toxoplasma gondii*, are distributed throughout the world.

This special issue compiles a series of ten original contributions and two comprehensive reviews that, while not being a complete representation of the field, is an important mixture of multifaceted knowledge that we have the pleasure of sharing with the readers. These articles cover relevant aspects of the biology and interaction between host cells and species such as* Trypanosoma cruzi, Trypanosoma brucei, Toxoplasma gondii, Plasmodium falciparum, Trichomonas vaginalis, Entamoeba histolytica, *and* Blastocystis *species.

O. Sheriff et al. use* Trypanosoma brucei* as a model to analyze the dynamics of the subpellicular microtubules visualized by inducible YFP-*α*-tubulin expression, particularly during new flagellum/flagellum attachment zone (FAZ) biogenesis and cell growth. Cytoskeleton modifications at the posterior end of the cells were also observed using the microtubule plus-end binding protein EB1, particularly during mitosis. The results suggest an intimate connection between new microtubule formation and new FAZ assembly.

C. M. Batista et al. describe the acquisition and characterization of a monoclonal antibody that recognizes cruzipain, the major cysteine proteinase found in* Trypanosoma cruzi*, especially in the reservosome, a special organelle of the endocytic pathway of this protozoan.

S. Cheemadan et al. describe the analysis of the role of calcium signaling in the transcriptional regulation of the apicoplast genome of* Plasmodium falciparum*. The transcriptional responses of this protozoan to two calcium ionophores were analyzed, as was developmental arrest in the schizont stage. In addition, a decrease of steady state mRNA levels was observed in essentially all transcripts encoded by the apicoplast genomes of cells treated with the ionophores. In addition, the apicoplast, a nuclear encoded protein with a calcium-binding domain, was identified and localized.

J. S. Calla-Choque et al. analyze gene regulatory processes at the transcriptional and posttranscriptional levels, mediated by the iron concentration in* Trichomonas vaginalis*. A protein was identified and designated as TvACTN3, the cytoplasmic protein that specifically binds to hairpin RNA structures from trichomonads when the parasites are grown under iron-depleted conditions. Thus, TvACTN3 could participate in the regulation of gene expression by iron in* T. vaginalis*.

C. Ximénez et al. describe the comparative analysis of the role of calreticulin (CRT) found in pathogenic* Entamoeba histolytica* and nonpathogenic* Entamoeba dispar* species that interact with human C1q, inhibiting activation of the classical complement pathway. CRT and human C1q are shown to colocalize in cytoplasmic vesicles located near the surface membrane of the parasite. The level of expression of CRT was analyzed in situ in lesions associated with amoebic liver abscess in the hamster, and the results suggested that CRT may modulate some functions during the early moments of the host-parasite relationship.

J. Pacheco-Yepez and coworkers analyze the molecular mechanisms involved in formation of amebic liver abscess induced by* Entamoeba histolytica*. Also investigated was the importance of peroxynitrite (ONOO^−^), both as the main agent of liver abscess formation during amebic invasion and as an explanation of the superior capacity of amebas to defend themselves against this toxic agent through the peroxiredoxin and thioredoxin systems.

Z. Wu et al. describe analysis of the interaction process of* Blastocystis*, an emerging protistan parasite colonizing the human intestine, with the polarized human colonic epithelial cell line Caco-2. It was shown that the protist induces apoptosis of the epithelial cells by activating host cell caspases 3 and 9 but not 8.

K. D. Cruz et al. present experimental evidence that lipid rafts from host cells (macrophages and epithelial cells) play some role in the process of host cell invasion by the pathogenic protozoan* Toxoplasma gondii*.

M. L. Chiribao et al. report a study of the interaction of* Trypanosoma cruzi* with epithelial cells in vitro where up to 1700 significantly altered genes, regulated by the immediate infection, were identified. This indicates that host cells are reprogrammed by* T*.* cruzi*, which affects cellular stress responses (neutrophil chemotaxis and DNA damage response), a great number of transcription factors (including the majority of NF-B family members), and host metabolism (cholesterol, fatty acids, and phospholipids).

M. R. Garcia-Silva et al. further analyze the role played by small RNAs found in microvesicles derived from the endocytic pathway and secreted into the extracellular medium by the pathogenic protozoan* Trypanosoma cruzi*. A large set of host cell genes were reportedly expressed upon incorporation of* T. cruzi*-derived extracellular vesicles, modifying the host cell cytoskeleton, extracellular matrix, and immune response pathways.

P. Florentino et al. review, from a historical perspective, how advances in microscopic imaging contributed to understanding the* Leishmania *spp. and* Trypanosoma cruzi* host-parasite relationships.

R. F. S. Menna-Barreto and S. L. de Castro review the pivotal role played by the mitochondria of the protozoan family Trypanosomatidae on oxidative stress and bioenergetics. These metabolic processes constitute an important target for the development of new drugs against these parasites, particularly if we can better comprehend mitochondrial oxidative regulation processes.

*Marlene Benchimol* *Juan C. Engel* *Kevin S. W. Tan* *Wanderley de Souza*

